# Treatment (Not Preventive) of Puerperal Fever

**Published:** 1889-02

**Authors:** Charles Warrington Earle

**Affiliations:** Professor of Diseases of Children and of Clinical Medicine, Woman’s Medical College; 535 Washington Boulevard


### THE TREATMENT (NOT PREVENTIVE) OF PUERPERAL FEVER.

BY CHARLES WARRINGTON EARLE,

PROFESSOR OF DISEASES OF CHILDREN AND OF CLINICAL MEDICINE, WOMAN'S MEDICAL COLLEGE.

The remark has been made that in consequence of the general use of antiseptics in Germany, there is no opportunity for studying septicæmia—that the disease in fact is vanished. To such an extent is this true that in some of the prominent journals devoted to obstetrics there is scarcely an allusion to the treatment of puerperal septicæmia during the past three years.

But in a certain percentage of cases infection has taken place. The chill, increased temperature, and the pelvic distress are all, or partly, present. Some way, either from the doctor, the nurse, or the patient herself, the infectious element has gained lodgment in the parturient's system.

Apart from these three sources from which the infectious material is usually furnished, a careful study of the subject leads me to believe that sometimes the condition which we have heard so ably discussed this evening, may arise from some old suppurating point hitherto not discovered, and in this way certain cases of puerperal fever, the origin of which it is extremely difficult to understand, is explained.

For instance, supposing a woman has an old pyosalpinx, which has remained quiet for months, and possibly years, the traumatism and pressure incidental to parturition, may, it appears to me, relight this process, and be a cause for either local or general infection. But whatever the cause, the phenomena of puerperal fever are present. Dread it, as we all do, it will come to us, not that it may be in our own practice, but to those who seem to delight in rejecting methods which almost insure immunity against it. In hospitals, which in olden times were justly regarded the hot-beds for puerperal fever, we find but few cases. An epidemic of puerperal fever in a modern hospital would at once be regarded as proof that some one had blundered. We must still be prepared to fight it in private practice. How shall we treat it? Mainly by the following processes and agents:

(1.) The vaginal douche and antipyretics.

(2.) The intra-uterine irrigation and antiseptics.

(3.) Curettement and antiseptics.

(4.) Drugs.

(5.) Surgery.

The following tissues and organs, according to the classification of Spiegelberg, must receive the local and general treatment. Not any two cases can be treated alike. Sometimes it must be a local remedy, at other times the removal of infective detritus. In other cases surgery, and in others nothing but drugs, from which we in many cases see absolutely no effect:

1. Inflammation of the genital mucous membrane—endocolpitis, and endometritis.

*a.* Superficial.

*b.* Ulcerative (diphtheritic.)

2. Inflammation of the uterine parenchyma, and of the subserous and pelvic cellular tissue.

*a.* Exudation, circumscribed.

*b.* Phlegmonous, diffused; with lymphangitis, and pyaemia (lymphatic form of peritonitis.)

3. Inflammation of the peritoneum covering the uterus and its appendages—pelvic peritonitis, and diffused peritonitis.

4. Phlebitis uterina, and para-uterine, with formation of thrombi, embolism, and pyæmia.

5. Pure septicæmia—putrid absorption.

If we have the first condition, a superficial vaginitis or endometritis, we should use local disinfection, iodoform, boracic acid, and, possibly, for the endometritis, the intra-uterine douche followed by the iodoform bacillus. The deep form of ulceration (and, possibly, diphtheritic) must be met with local cleanliness and applications of some destructive agent, such as the chloride of zinc in proper solution, the bichloride of mercury, with applications of iodoform or boracic acid. Every diphtheritic patch must be touched with the local application, and the parts thoroughly washed out with an antiseptic fluid. Patches which can be reached should be packed with iodoform gauze.

In the second condition, inflammation of uterine tissue proper, with circumscribed exudate, we should satisfy ourselves that no local point of infectious material remains, and treat the patient by insisting that she remain in bed till all pain subsides, meet all drug indications that follow, and follow with hot vaginal douches for a long period, to which should be added a generous diet and tonics. If the para-uterine tissues are involved (connective tissue immediately adjacent to the uterus) we must administer anodynes, use fomentations, and relieve the symptoms produced by exudates in the vicinity of the bladder and other pelvic organs.

With the use of the above treatment, with hot douches, the mild and uncomplicated cases disappear, and the formation of pus is the exception. Sometimes, however, we have a hard tumor remaining for a long time, which is usually dissipated by counter-irritation, hot injections, good diet, and tonics.

Pelvic peritonitis and general metropertitonitis are among the fatal forms of puerperal fever, although the first will usually recover if the source of infection is not too pronounced. General peritonitis is extremely dangerous, and in the majority of cases, under the old règime, absolutely fatal. In addition to the anodynes, which have for years been our sheet-anchor, and which in some cases are pushed in enormous doses, the question of drainage will certainly arise. It is an open question with me to-day whether such large doses of opium, producing constipation and retention within the system of germs, is the best treatment. The abdominal cavity will be found filled with brownish or green fluid, and without its removal any treatment will be futile. It is in the first stage of this form of the disease that cocaine for vomiting, rectal injections of hot water for the extreme thirst, and saline laxatives as local depletants, should be used.

The treatment by salines or laxatives is based upon actual observations and clinical experience. It has been known for a long time that some of the inferior animals poisoned by septic matter recover after a long and persistent diarrhœa, and the usefulness of laxatives in puerperal fever has been strongly recommended by Latour Seyfert and Breslau (Schroeder). The best hope of eliminating the poison comes from this method, and not only this, but their administration reduces the fever, and the pain becomes less. Sometimes nature does better than the doctors, and a diarrhœa is set up at the commencement, which persists until convalescence is established. If diarrhœa is present without laxatives, this is all that is needed. If not present, and the administration of salines does not produce it, the prognosis is bad. Eulenberg assures us that no fear need be entertained that the copious discharges from the intestinal canal will weaken the patient, or that there will be any trouble in checking them at the proper time. The great weight of authority at this day is in favor of this treatment.

Among the remedies to bring about and continue this method of cure may be mentioned the bitter waters, caster oil, jalap and calomel, Hunyadi water, and perhaps best of all, magnesia sulph. These are to be given in such doses and at such times as will influence and keep up the depletant effect.

The fourth form of puerperal fever in the classification I have chosen is *uterine phlebitis*, with or without metastatic abscesses. It will not be necessary for me to trace for this audience the history of a phlebitis, sometimes making its appearance as a painful spot in the lower limb, with increased swelling and pain, the indurated blood-vessels, the irregular chills, and indications of pus-points in different parts of the body. It is not new to class this as one form of puerperal fever, although there may be some who still believe in the old milk-leg theory of the ancient nurses and grandmothers.

The treatment is simple and trifling in some cases, recovery is rapid; the infection is only slight and local. In others absolutely nothing seems to do our patients good, and we must be content with tonic and stimulant treatment, with a generous diet to sustain the strength of the patient until the infection is worn out, which, after all we can do, after long weeks of suffering, will in many cases cause the patient to succumb. This infection takes place at the placental site, and it comes on so slowly that the most experienced sometimes will not detect it. The poison is distributed to such remote parts of the body that we are disarmed at the very outset. We cannot reach with any known remedy the point of lodgment of emboli in distant organs; we can only destroy what remains of the infectious material at the original point of entrance, if we can happily discover it, and try to sustain the strength of the patient till nature effects a cure.

It is hardly worth while for me to say anything in regard to a treatment for pure septicæmia. Sometimes the symptoms of blood-poisoning are so intense that the patient will die without any local lesions. What can be done has either been brought out in what has been said regarding general medication, or will be noted in the remarks on laparotony.

In all of these different manifestations, however, there underlie certain general principles which we should be ready to adopt. The first is vaginal cleanliness and antipyretics.

I know that in a few cases patients do not seem to do as well after even vaginal douches, but in the main it is because they are improperly given. Sometimes cleansing the vagina a single time will reduce temperature and alter the entire character of a threatened puerperal attack. If this does not reduce the temperature, then the next procedure should be an intra-uterine douche. This should be done with the utmost care and with a full realization of the dangers which sometimes take place. One about to give an intra-uterine douche should know that accidents sometimes happen from entrance of air into the uterine sinuses, and that sometimes a hæmorrhage is produced. We should know, too, that the injecting-fluid may be introduced into the general circulation by the introduction of the injection-tube into the mouth of one of the large sinuses; that convulsions and pain may be produced, and that fluid may be forced into the peritoneal cavity through a Fallopian tube. If time permitted I could demonstrate that but little reason really exists for apprehending any of these accidents. Notwithstanding these dangers, however, it seems to me that the uterine douche is justifiable, and that in some conditions nothing will take its place. It is absolutely useless to administer drugs to a patient who is suffering from an infection from a retained placenta or from shreds of membrane, or from an old decomposing clot, or from retained lochial discharges. These must be removed, and the cavity must be kept clean. To be sure, we do not alter or change in any degree the infectious material which has been already absorbed into the system, but by washing away repeatedly these new sources of infection, we prevent the system from being re-loaded time after time by this poisonous stuff.

An intrauterine injection cannot be done with a half-ounce syringe and a goose-quill. One must realize the dangers, have the proper instruments, and know *how* to use them. The various steps are:

1. Clean the outside of the patient around the vulvar orifice.
2. Thoroughly cleanse the vagina.
3. Give the intrauterine douche.
4. Introduce iodoform bacillus.

Always have the stream of water running when the injection-tube is introduced into the uterine cavity. If the temperature does not fall in the course of a few hours, and there is a lochial discharge which is offensive, showing beyond a doubt that there is decomposing matter which has been infected in some way still remaining in the uterine cavity, it appears that now the time has come either for repeated intrauterine douches or curettement. I am well aware that there are cases which neither the vaginal, nor the intrauterine douche, will effect. Not every parturient canal is a traumatic channel—the poison is often elsewhere. There are cases of puerperal fever in which, it appears to me, it is impossible to discover the exact *cause* of the infection or *its location*. If the infection has localized itself along the side of the uterus, and makes its appearance as a pelvic cellulitis, or as an inflammation in the broad ligaments, of course no good comes from repeated vaginal or intrauterine injections, but until we can demonstrate the localization of this poison the treatment by douches will give us the best results. Whatever the cause, the first thing we should do is to properly cleanse the vaginal tract and search for some point of infection in this location. If a perineum has been restored and sutures of any kind been used, unless union is perfect, which will be rare indeed if infection has taken place, it appears to me that at this point very careful search should be made, and in addition to giving a vaginal douche, in my judgment the patient will be placed in a safer condition if these stitches are taken out and the old lacerations thoroughly cleansed.

In regard to *vaginal douches*, I desire to say that I do not believe in doing *anything* to the parturient woman unless there are indications. But if a laceration has taken place, which has been closed by one, two or three stitches, at least a daily vaginal douche with either carbolized or bichloride water should be ordered.

Among the drugs recommended in the treatment besides those I shall mention incidentally in connection with other methods of cure, I shall speak of quinine, antipyrin, veratrum viride, turpentine, opium, iodoform, alcohol and hot baths. The use of the cold water coil and constant irrigation should also be mentioned.

Quinine is undoubtedly an excellent remedy, and may be used either in antipyretic or tonic doses. Sometimes we cannot do better than to employ this remedy throughout the course of a puerperal fever. Antipyrin has been useful in reducing temperature, but its liability to produce shock makes me careful in its use in a disease which in itself severely taxes the strength of the patient. In the German Gynæcological Society of 1886 antipyretics were discarded, while the advantages of hot baths and alcohol were reiterated. Professor Fordyce Barker will be remembered as the particular advocate of veratrum viride, a remedy most useful in some other diseases, but to me contra-indicated here as other depressants are.

Turpentine has been useful externally, and has been administered by stomach—indeed, no one other remedy seems to do quite as well when the patient has a red tongue and some other symptoms simulating a typhoid condition. It has been recommended recently by Thomas Madden in every form of puerperal fever.

Iodoform has been given internally in one case with good results.

The salicylates are also recommended, and theoretically they should do good in certain cases.

I have said all regarding the opium-treatment that seems to be necessary. It appears to me that in the light of recent investigations, any remedy which persistently constipates, presenting local depletion, is contra-indicated.

In the Transactions of the Ninth International Medical Congress, Dr. Nelson of this city speaks of the beneficial results from the sulpho-carbonic acid treatment. As far as I know, this remedy has not been used to a sufficient extent so that we can regard it as authoritative.

The use of alcohol in all forms of septicæmia is so well established that I need say nothing regarding its results.

The reduction of the temperature by cold water, used by means of the coil placed over the abdomen, is one of the best agents to combat this symptom, which I have ever employed. Kibbee's cots, by which a large extent of coil through which cold water is introduced, has also been recommended by some.

Say everything that we can in regard to drugs, there is nothing which has been brought prominently to the front during the past one or two years. Without other means than internal medicine to combat some forms of puerperal diseases, we are almost absolutely powerless, and we stand by the bedside of our patients watching them as they come under the influence of the infectious material, and gradually failing until death closes the scene.

In the local treatment of puerperal septicæmia, says a recent author, "antiseptic intrauterine injections still hold their deserved preëminence. Bokelmann considers them indicated when forty-eight hours after birth the temperature rises to 101.5° or 102.2° Fahr., with frequent pulse without a recognizable cause for it; (2) when fragments of placenta or membranes remain in the uterus as a cause for disturbance; and (3) when symptoms of infection of the endometrium are present."

After vaginal injections and disinfection, followed by the intrauterine douche with the iodoform bacillus, if evidence still remains that decomposing clots or intrauterine débris of any kind are yet present, we should resort to curettement. I cannot see that the character of the pulse or any other symptoms can have much weight in causing us to decide in this manner. If there is evidence that any extraneous substance which has been infected is still left in the uterus, it appears to me it should come out. A stream of hot water cannot in some cases detach it. It still remains to again and again furnish infection, which is again and again to overpower the system. Take it away, not with a sharp instrument, not with manipulations which involve going through the uterine walls, but by first doing everything which has been suggested in the operation for the uterine douche, then curette, then give another douche, and follow with the iodoform bacillus. Such an operation as this carried out carefully, and with all antiseptic details, will sometimes change the prognosis of a puerperal fever with remarkable rapidity. The fact that some one not skilled in the use of the instrument has perforated the walls of the uterus is no argument against the operation.

I have not time to enter into the treatment of all of the complications and sequelæ which sometimes arise after a case of puerperal fever. It must suffice to say in regard to local abscesses, for instance, if they are within easy reach, it is perhaps as good practice as any to aspirate. We know that sometimes after aspiration the abscess disappears, does not refill, the temperature goes down, and the patient makes a good recovery. If, however, the abscess-cavity does refill, it must be found and properly drained. Sometimes this can be done through the cul-de-sac of Douglas, at other times by opening the abdomen and draining from this point. In some cases of doubt, I have seen the abdominal cavity opened, and the abscess found; the fact is demonstrated that the sac cannot be either extirpated or brought to the surface of the abdominal wound, and consequently drainage must take place through the vagina, the abdominal wall being closed.

The relation of pyosalpinx to puerperal fever, and what shall be done with large collections of pus within the abdominal cavity, which sometimes follow in the course of puerperal peritonitis, is at this time receiving more than the usual amount of attention. Dr. Baldy, of Philadelphia, one month after labor has opened the abdomen and removed the right tube, which was distended with pus, and the patient, although in a very critical condition when the operation was done, rapidly recovered.

The treatment of metroperitonitis, or purulent peritonitis, has also undergone a great change. These cases have been regarded as almost hopeless. We have stood by the bedside of a woman with an abdomen enormously distended, and with all the symptoms of septicæmia, with undoubted evidences of pus in the cavity, and allowed her to die without opening the abdominal cavity. This in the future will not do. There is testimony, which is increasing every day, that if we allow such a condition of things to remain without, at least, making an effort to save our patient by drainage we fall short of doing our duty.

One of the most important advances in obstetric surgery during the past two or three years has been made by Tait, who for some time has been advocating abdominal incisions, and cleansing of the peritoneal cavity in such cases. During these years he has been making appeals to the practitioners in his neighborhood to be allowed to perform such operations, which at this time have been done in a few cases with excellent results. An abdominal incision is made, the foul fluids cleared out, a drainage-tube inserted, and the wound closed.

Greig Smith makes the suggestion that in treating cases of suppurative inflammation of the peritoneum the intestines should be kept floating in a hot antiseptic lotion. The fluid which he recommends is the hot boro-glycerine solution, which is repeatedly passed in through a drainage-tube and allowed to remain in the cavity for a short time. The fluid should be at least 102° Fahr. This operation with its technique is suggested by two of our greatest operators, and all details are not worked out. The absolute indications and the results must be decided by future research. The results cannot possibly be more discouraging than from our past treatment.

*535 Washington Boulevard.*

